# A Model for Remote Depth Estimation of Buried Radioactive Wastes Using CdZnTe Detector

**DOI:** 10.3390/s18051612

**Published:** 2018-05-18

**Authors:** Ikechukwu Kevin Ukaegbu, Kelum A. A. Gamage

**Affiliations:** 1 Engineering Department, Lancaster University, Lancaster LA1 4YW, UK; 2 School of Engineering, University of Glasgow, Glasgow G12 8QQ, UK; Kelum.Gamage@glasgow.ac.uk

**Keywords:** remote depth profiling, attenuation model, radiation detection, radioactive contamination, radiological characterisation, nuclear wastes, nuclear decommissioning, cadmium zinc telluride

## Abstract

This paper presents the results of an attenuation model for remote depth estimation of buried radioactive wastes using a Cadmium Zinc Telluride (CZT) detector. Previous research using an organic liquid scintillator detector system showed that the model is able to estimate the depth of a 329-kBq Cs-137 radioactive source buried up to 12 cm in sand with an average count rate of 100 cps. The results presented in this paper showed that the use of the CZT detector extended the maximum detectable depth of the same radioactive source to 18 cm in sand with a significantly lower average count rate of 14 cps. Furthermore, the model also successfully estimated the depth of a 9-kBq Co-60 source buried up to 3 cm in sand. This confirms that this remote depth estimation method can be used with other radionuclides and wastes with very low activity. Finally, the paper proposes a performance parameter for evaluating radiation detection systems that implement this remote depth estimation method.

## 1. Introduction

Wastes generated during the nuclear fuel cycle can end up in the soil, resulting in large-scale land contamination. This is the case in the beaches of Dounreay in Northern Scotland where wide-spread radioactive soil contamination has been reported [1,2]. This was caused by the so-called Dounreay particles resulting from the processing of the fuels from the Material Test Reactor at the Dounreay nuclear facility. These particles consist mainly of Caesium-137 (Cs-137) fuel fragments buried at depths less than 40 cm and extending over an area of about 200,000 m^2^. Other sources of shallow subsurface radioactive contamination include: leaks from waste pipes [3,4] and radioactive fallout from nuclear tests [5]. The characterisation of these subsurface wastes has continued to be a major nuclear decommissioning challenge [6]. This is mainly because of the difficulty in estimating the depth of penetration of these contaminants without having recourse to destructive methods such as logging or core sampling [7,8]. Furthermore, reported non-intrusive depth estimation methods for such wastes are either based on empirical models [9,10] or are limited to a maximum depth of 3 cm [11,12].

Consequently, a novel remote depth estimation method for buried waste was recently developed [13]. This method is based on an approximate three-dimensional linear attenuation model that makes use of multiple radiation measurements obtained from the surface of the material in which the radioactive contaminant is buried. The results from simulation showed that the method is able to estimate the depth of radioactive point sources buried up to 40 cm in both sand and concrete. Furthermore, results from initial experiments using an organic liquid scintillator (EJ-301) from Eljen Technologies (Sweetwater, TX, USA) and a high-speed multichannel analyser MFAx1.3 from Hybrid Instruments Limited (Lancaster, Lancashire, U.K.) achieved a maximum detectable depth of approximately 12 cm at an average count rate of 100 cps, where the average count rate is defined as the average of the count rates at each depth when the detector is located axially with the source. These preliminary results indicate that improved results can be obtained using a radiation detector with a better gamma spectral response. This is because the gamma spectral response of the EJ-301 scintillator is limited to the Compton continuum.

Therefore, this paper presents improved results from the depth estimation method using a Cadmium Zinc Telluride (CZT) detector. The CZT is a semiconductor detector that is well known for its good spectral response at room temperature [14]. Consequently, it is widely used in the characterisation of nuclear materials in fields such as nuclear safeguarding and decommissioning [15,16]. The paper also reports on the effect of data acquisition time on the depth estimation performance of the method and proposes a performance parameter for evaluating systems that will implement the depth estimation method. The next section gives a detailed description of the materials and methods used in the research. The experimental results are presented and discussed in Section 3, while the conclusions and future directions are presented in Section 4.

## 2. Materials and Methods

### 2.1. The Approximate Three-Dimensional Linear Attenuation Model

**Figure 1:**
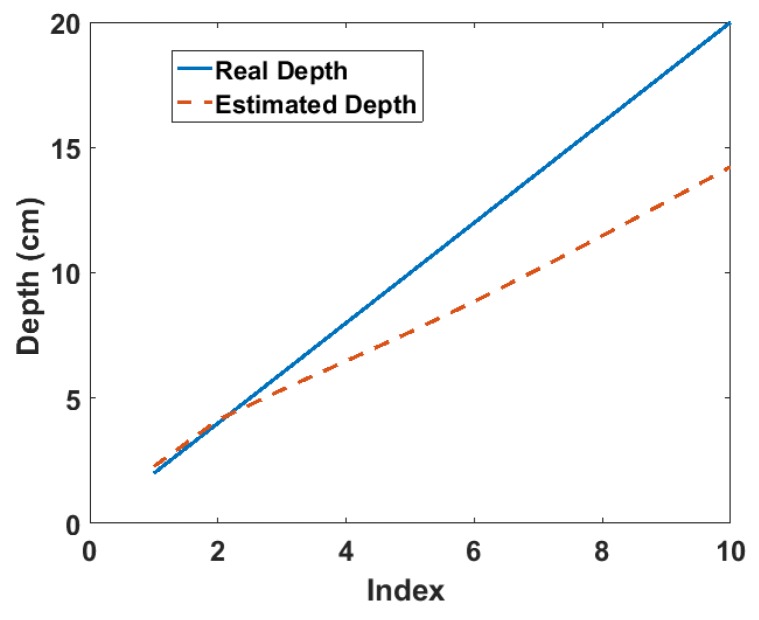
Estimated depth from the simulation of the Cs-137 point source buried in sand [13]. The index refers to the position of each depth value in the depth array, i.e., 1 = 2 cm, 2 = 4 cm, 3 = 6 cm, etc.

Let $I_{\left(x,y,z\right)}$ be the radiation intensity measured at any position (x,y) on the surface of a material volume in which a radiation source is buried at depth *z*. The ratio of this intensity to that measured from a reference position (i.e., (x,y)=(0,0)) on the same surface is given by:

(1) $$log_{e}\left(J_{\left(x,y,z\right)}\right)\approx -\frac{\mu }{2z}\left(x^{2}+y^{2}\right)+log_{e}\left(K_{\left(x,y,0\right)}\right)$$

where $J_{\left(x,y,z\right)}=\frac{I_{\left(x,y,z\right)}}{I_{\left(0,0,z\right)}}$, μ=linearattenuationcoefficient and $K_{\left(x,y,0\right)}=\frac{I_{\left(x,y,0\right)}}{I_{\left(0,0,0\right)}}$. Equation (1) is the approximate three-dimensional linear attenuation model derived in a previous work [13]. It expresses the ratio of the intensity measured at any position on the surface of the material volume to that measured at the reference position on the same surface. Furthermore, it can be deduced that the approximate depth of the source can be estimated from the gradient of the model. The gradient can be obtained by fitting a linear polynomial to the graph of the model for a set of spectra acquired from multiple positions on the surface of the material volume in which the source is buried. The simulation result of this procedure for Cs-137 buried in sand at depths from 2 cm to 20 cm at 2-cm intervals is shown in Figure 1. The deviation observed at increasing depth is as a result of the approximation made in the derivation of Equation (1). The details of the model derivation and a comprehensive analysis of the simulation results have been reported in [13].

### 2.2. Experiment

**Figure 2:**
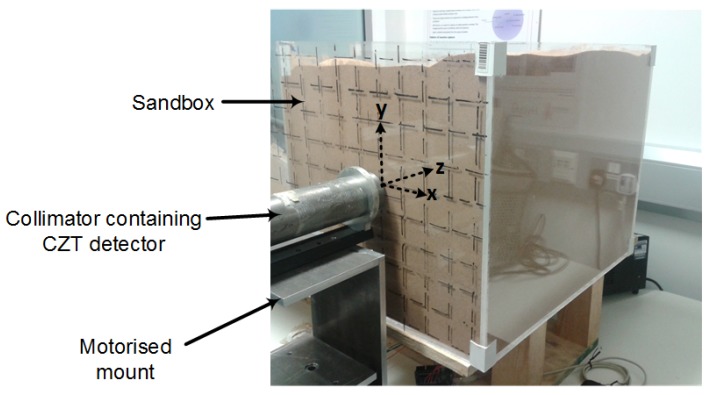
Setup for the experiment. The Cs-137 point source was placed at varying positions along the *z*-axis using a pipe that protruded behind the box, while the intensity was measured at the grid positions marked on the surface of the sandbox. CZT, Cadmium Zinc Telluride.

The experimental setup (Figure 2) consisted of a sandbox filled with sand in which the radiation source was placed at varying distances from the front surface. The walls of the box were 0.8 cm thick and were constructed using acrylic plastic sheets. The density of the sand was 1.66 g cm^−3^, and the weight fractions of its composite elements obtained using Scanning Electron Microscopy (SEM) are shown in Table 1. The radiation source was attached to one end of a plastic pipe whose other end protruded behind the box. This was used to vary the position of the source along the z-axis. The detector was placed inside the cylindrical tungsten collimator shown in Figure 2 so that only gamma rays within the detector’s field of view were detected at each *x*-*y* position. The collimator was 1 cm thick, 25 cm tall and had an internal diameter of approximately 4 cm. Furthermore, the collimator was attached to a motorised mount for automated positioning of the detector at each specified position on the front surface of the sandbox.

In order to acquire the data, the total scanning area was set to 28×28 cm^2^, which was divided into 4×4 cm^2^ grids. The size of the grids were chosen to be approximately equal to the internal diameter of the collimator. The radiation source was then positioned at distances (i.e., depths) varying from 2 cm to 20 cm at 2-cm intervals from the front of the sandbox. At each depth, the detector was moved across the scanning area, and the spectrum of the buried source was measured at each grid, thereby yielding a total of 49 spectra per depth.

### 2.3. Spectrum Acquisition and Preprocessing

**Figure 3:**
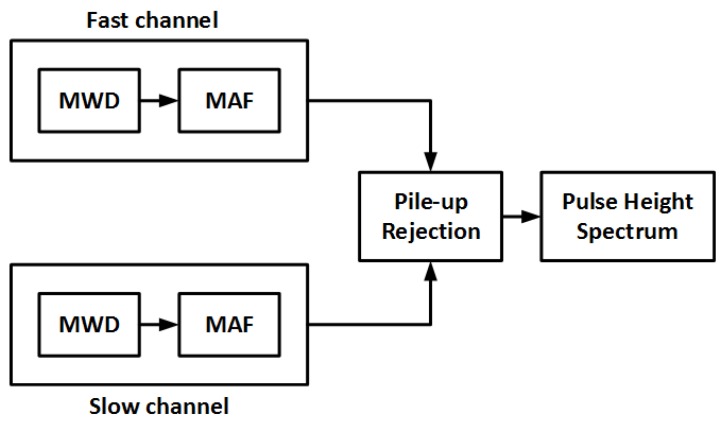
Pulse height spectrum analysis algorithm used in the experiment where MWD is the Moving Window Deconvolution and MAF is the Moving Average Filter.

The detector used in the experiment was the CZT/500S detector from Ritec (Riga, Latvia). It is a quasi-hemispherical CZT detector with a sensitive volume of 0.5 cm^3^ and is enclosed in a cylindrical casing of diameter 2.2 cm and height 3.3 cm. Therefore, it was able to fit inside the collimator used in the experiment. The output from the detector was connected to a charge-sensitive low noise preamplifier (PA101C also from Ritec), and the output pulses were sampled by an oscilloscope (sampling rate = 500 kS/s) controlled by a Personal Computer (PC). After digitisation by the oscilloscope, the pulses were then transferred to the PC via Ethernet for Pulse Height Analysis (PHA). The stages of the PHA are as shown in Figure 3. It consists of a fast and a slow processing channels whose outputs are used by the pile-up rejector to estimate the height of suitable pulses. The details of the PHA are discussed as follows.

(a) Moving window deconvolution:

The Moving Window Deconvolution (MWD) is an efficient filter proposed by Georgiev et al. [17] for the retrieval of the amplitude of the step pulse from the output of the preamplifier. The charge collected when a photon strikes the CZT crystal creates a fast rising step in the output of the preamplifier with an amplitude that is proportional to the amount of charge collected. However, the step decays exponentially at a rate determined by the time constant of the preamplifier. This delay of the signal to return to baseline prevents the accurate measurement of the amplitude of subsequent voltage steps. However, since the output from the preamplifier is a convolution of the charge distribution function and the impulse response of the preamplifier, deconvolving this output signal will enable the reconstruction of the original charge distribution function while eliminating poles from the preamplifier transfer function [17]. The MWD performs this deconvolution in a moving time window and is given by:

(2) $$MWD\left(i\right)=X\left(i\right)-X\left(i-M\right)+\frac{1}{\tau }\sum_{j=i-M}^{i-1}X\left(j\right)$$

where X(i) is the value of the signal at the *i*-th sample, *M* is the window size and τ is the preamplifier time constant in units of sample time. It can be observed that the MWD is a differentiator followed by an integration term that compensates for the exponential decay using the time constant of the preamplifier.

(b) Moving average filter:

The moving average filter is an optimum constant weight smoothing filter suitable for reducing random noise. It was applied to the output of the MWD so as to reduce the noise level without affecting the energy resolution [18]. It is given by:

(3) $$MAF\left(i\right)=\frac{1}{L}\sum_{j=i-L}^{i-1}MWD\left(j\right)$$

where *L* is the filter length. The value of *L* in relation to the MWD window size *M* determines the output pulse shape. For instance, L<M results in trapezoidal shaping, while L=M results in triangular shaping.

(c) Pile-up rejection:

Pile-up is caused by two or more events occurring within the duration of the length of the shaping filter (i.e., the window size of the MWD). This causes the events to be processed into a single pulse resulting in the wrong estimation of the pulse amplitude. The need to reduce pile-ups is usually in conflict with the need to ensure complete charge collection. This is because while long shaping times increase the probability of complete charge collection, they also increase the occurrence of pile-ups, especially at high count rates. Pile-up rejection was implemented in PHA by having two processing channels: a slow channel with a longer shaping time for increased probability of complete charge collection and a fast channel with a shorter shaping time for resolving closely occurring events. Therefore, any pulse from the slow channel with more than one pulse within the same duration in the fast channel is rejected as a pile-up.

**Figure 4:**
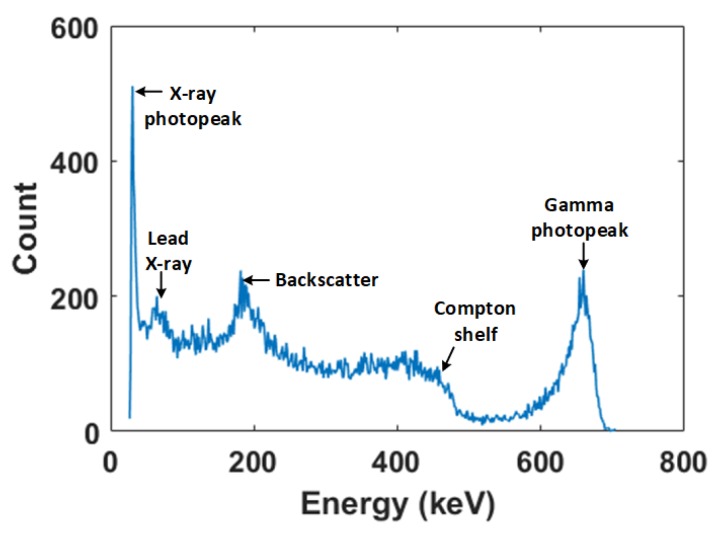
Cs-137 spectrum from the Pulse Height Analysis (PHA) algorithm after 50,000 counts.

The PHA algorithm was implemented in MATLAB (Natick, MA, USA) and used to process the pulses transferred from the oscilloscope. The Cs-137 spectrum obtained after 50,000 counts using long and short filter shaping times of 10μs and 7μs, respectively, is shown Figure 4. It can be observed that all the key features of the Cs-137 gamma spectrum can be clearly identified.

#### Photo-Peak Fitting

**Figure 5:**
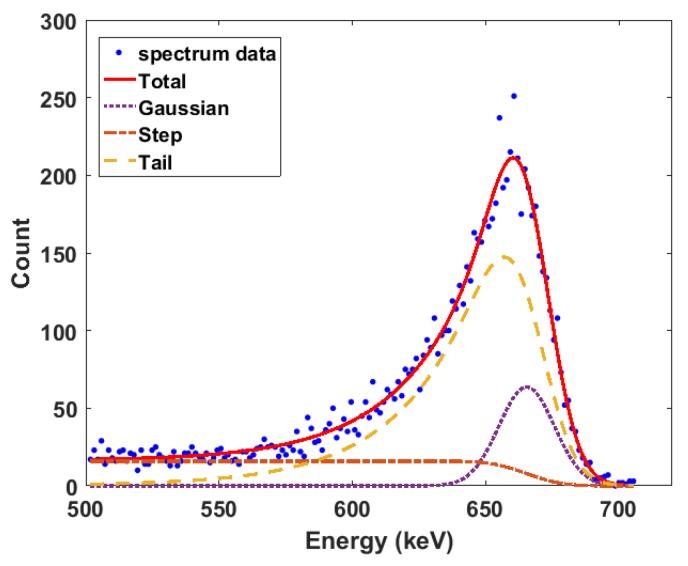
Cs-137 662-keV photopeak fitting using Equation 4.

One of the problems with the spectrum produced by CZT detectors is the elongated low-energy tail of the photo-peak. This is because of incomplete charge collection caused by early carrier recombination due to low hole mobility within the CZT crystal [19]. Consequently, the obtained spectrum photo-peaks are often asymmetric and cannot be adequately described by conventional Gaussian functions. Therefore, Montreau et al. [19] proposed a more robust peak fitting function given as:

(4) F(i)=G(i)+S(i)+D(i)+B(i)

where *i* is the channel number, and *G* is a Gaussian function given by:

(5) $$G\left(i\right)=H_{g}exp\left[-{\left(i-i_{0}\right)}^{2}/2\sigma^{2}\right]$$

where $H_{g}$ is the amplitude of the Gaussian function, $i_{0}$ is the centroid and σ is the standard deviation. S(i) is a step function given by:

(6) $$S\left(i\right)=H_{s}H_{g}erfc\left[\left(i-i_{0}\right)/\sqrt[{}]{2}\sigma \right]$$

where $H_{s}$ is the height of the step. D(i) is an exponential tailing function described as:

(7) $$D\left(i\right)=H_{t}H_{g}exp\left[\left(i-i_{0}\right)/T_{s}\sigma \right]\times erfc\left[\left(i-i_{0}\right)/\sqrt[{}]{2}\sigma \right]+1/\left(\sqrt[{}]{2}T_{s}\right)$$

where $T_{s}$ is the inverse slope of the exponential tail. The last component of the fitting function B(i) represents background radiation; however, it can be neglected if background subtraction is performed before fitting the function to the photo-peak. Figure 5 shows the application of Equation (4) to the 662-keV photo-peak of the acquired Cs-137 spectrum. The contribution of each component of the fitting function to the accuracy and robustness of the fit can be observed. Therefore, this photo-peak fitting function was used to analyse the spectra obtained from the experiments after background subtraction.

## 3. Results and Discussions

### 3.1. Results for Caesium-137

**Figure 6:**
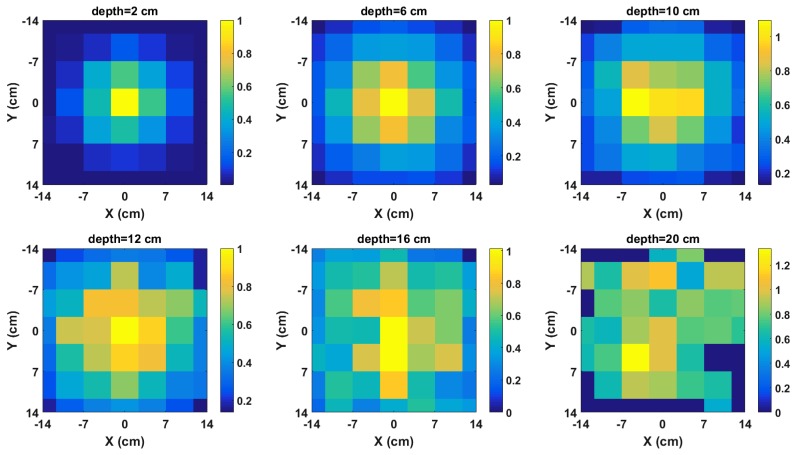
Normalised radiation images of Cs-137 buried in sand for selected depths.

The two-dimensional radiation image for some selected depths between 2 cm and 20 cm for a 329-kBq Cs-137 point source buried in the sandbox are shown in Figure 6. The pixel values of each image are the photon count at 662 keV of the spectrum acquired at that position on the front surface of the sandbox. In addition, the spectra were acquired using a scanning time of 25 min per *x*-*y* position. It can be observed that the images show an increasing defocussing of the intensity from the centre towards the edges as the depth increases. This shows that the distribution of the intensity across the image pixels contains information about the depth of the source. Furthermore, it can be observed that at the depth of 20 cm, the pixel intensities become randomly distributed. This is because of significant attenuation, which causes some pixels to have zero values. These zero-valued pixels represent positions where the photo-peak fitting function failed due to its inability to detect a peak. This distribution of the intensities across the image in addition to the decrease in the photon count due to attenuation are the two pieces of information exploited by this method to estimate the depth of the buried radioactive source.

**Figure 7:**
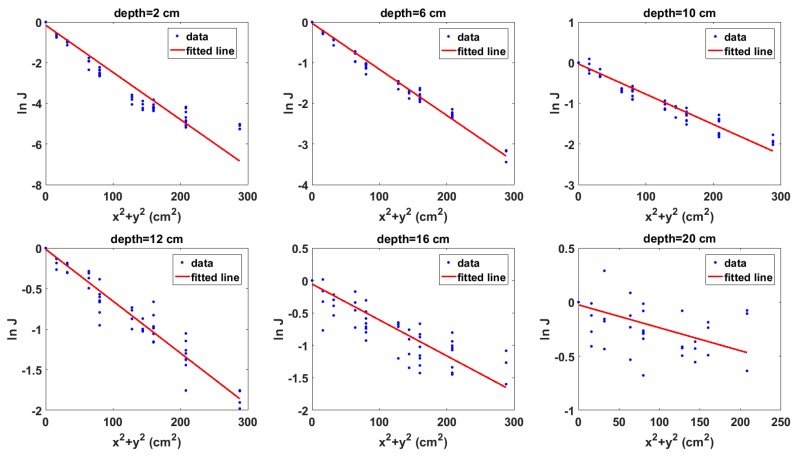
Graphs of the model for Cs-137 buried in sand for selected depths. (x,y) is the position of the detector on the surface of the sandbox, while *J* is the ratio of the intensity measured at each (x,y) to that measured at the centre of the sandbox surface.

The next step in the depth estimation process is the evaluation of the model (i.e., Equation (1)) using the radiation images. The graphs of the model for the same selection of depths whose images are shown in Figure 6 are shown in Figure 7. It can be observed that the graphs have negative gradients as predicted by the model. Furthermore, it can also be observed in the graphs in Figure 7 that the gradient of the fitted polynomial tends to zero as the depth increases. This shows that the model aptly preserves the attenuation behaviour of gamma rays in materials. In addition, it also implies that the gradient of the data points contains information about the depth of the source and that the quantity of depth information in the gradient decreases as the gradient value tends to zero where a zero gradient value means no depth information. However, it must be noted that zero represents an absolute limit because the reliability of the depth estimates will become significantly reduced even before the gradient value becomes zero.

**Figure 8:**
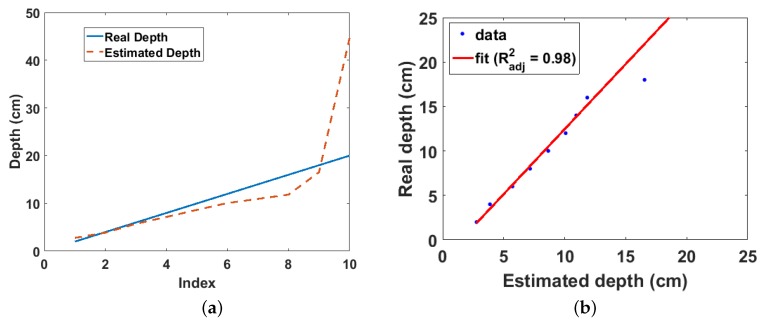
(**a**) Real and estimated depths for Cs-137 buried in sand. The index is the position of each depth value in the depth array, i.e., 1 = 2 cm, 2 = 4 cm, 3 = 6 cm, etc.; (**b**) Linear fit of real and estimated depth for Cs-137 buried in sand.

The depths of the source estimated from the gradient of the model are shown in Figure 8a. The linear attenuation coefficient μ at 662 keV for sand was calculated using the weight fractions in Table 1 and the mass attenuation coefficients published by the National Institute of Standards and Technology (Gaithersburg, MD, USA) [20]. It can be observed that the estimated depth approximates the real depth up to 6 cm well, after which the expected deviation from the real depth begins. As explained in [13], this deviation is a result of using only the first two terms of the binomial expansion in the derivation of the model. This deviation continues up to 16 cm, after which a slight upward jump can be observed at 18 cm. This slight jump at 18 cm indicates the depth at which the effects of attenuation begin to introduce errors to the estimate. This slight jump is followed by a complete divergence of the estimated depth from the real depth at 20 cm due to large errors in the estimate caused by significant attenuation of the gamma rays. This complete divergence in the estimated depth at 20 cm is corroborated by the complete defocussing of the radiation image at 20 cm (Figure 6) and the almost zero gradient of the fitted polynomial in the model graph also at 20 cm (Figure 7).

Figure 8b shows that there is a linear relationship from which the real depth can be predicted from the estimated depth up to 18 cm with an adjusted R-squared value of 0.98. A weighted linear regression was used for the polynomial fitting to limit the effect of the slight error in the estimate at 18 cm on the regression parameters. In addition, the estimate at 20 cm was not included in the polynomial fitting due to the large error in the estimate as already mentioned. The average corrected count rate obtained for this experiment was 14 cps due to the slow communication link between the PC and the oscilloscope. However, this result is a significant improvement compared to the result previously obtained using the EJ-301 scintillator [13], which achieved a maximum detectable depth of 12 cm with a significantly higher average count rate of 100 cps. In addition, this result also suggests that using the CZT detector with a commercial multichannel analyser rather than the improvised setup employed in this experiment, this depth estimation method can achieve a maximum detectable depth greater than 18 cm using less than 25-min per *x*-*y* position scanning time. This improved result is mainly due to the good gamma energy resolution of the CZT detector, which enabled the use of photon counts from the photo-peak in the depth estimation. Conversely, the experiment with the EJ-301 used photon counts from the Compton peak because the EJ-301 could only produce the Compton continuum of the gamma spectrum. Finally, this result confirms that while photon counts from any part of the spectrum can be used with the model, photon counts from the photo-peak will yield the best results.

#### Effect of Scanning Time

**Figure 9:**
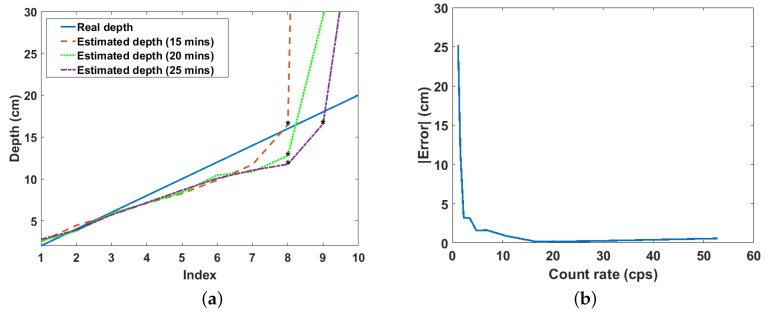
(**a**) Estimated depths for three different scanning times. The index is the position of each depth value in the depth array, i.e., 1 = 2 cm, 2 = 4 cm, 3 = 6 cm, etc.; (**b**) Exponential decrease of the absolute error in the estimated depth with increasing count rate.

The estimated depths for different scanning times per *x*-*y* position, namely 15, 20 and 25 min, are shown in Figure 9a. A gradual, but progressive improvement in the estimated depth at 16 cm and 18 cm can be observed as the scanning time increases (see the points indicated on the graph). This progressive improvement in the estimated depth results in the progressive restoration of the graph to the expected deviation pattern as the scanning time increases. The gradual rate at which the estimates improve with time indicates that this relationship is exponential. This is confirmed by Figure 9b, which is the graph of the absolute error in the estimate as a function of the count rate for the 20-min scanning time experiment. The decay rate of this graph indicates how quickly the error in the estimated depth decreases as the count rate increases, and it is independent of the scanning time. Furthermore, dividing this decay rate by the density of sand will make it also independent of the material in which the source is buried. This will result in a value that is dependent only on the efficiency of the instrumentation (i.e., detector and related electronics) used. Therefore, this value can be used as a parameter for evaluating and selecting appropriate instrumentation for field application of this remote depth estimation method.

The gradients and intercepts for the linear fit between the real and estimated depths for the three scanning times and those from simulation (reported in [13]) are shown in Table 2. The “Depths” column in the table refers to the range of depths over which the parameters were estimated. It can be observed that the values of these parameters are relatively constant and do not vary significantly with depth. This means that the values of these parameters can be assumed to be constant for any given material and gamma energy. Therefore, this method can be used to investigate radioactive wastes buried at any depth in a given material without the need for calibration. This is not the case with the empirical model proposed in [9,10], where new model parameters must be obtained in order to estimate depths outside the range of depths used to develop the model.

### 3.2. Results for Cobalt-60

**Figure 10:**
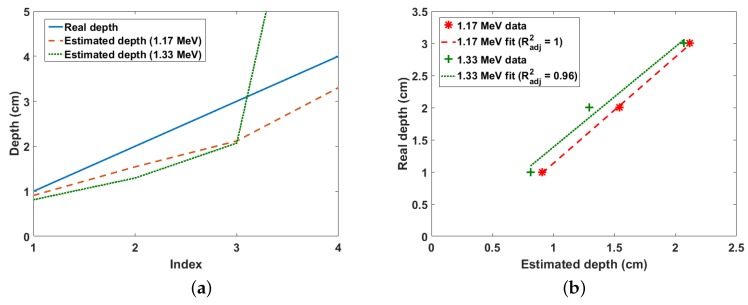
(**a**) Real and estimated depths for Co-60 buried in sand. The index is the position of each depth value in the depth array, i.e., 1 = 2 cm, 2 = 4 cm, 3 = 6 cm, etc.; (**b**) Linear fit of real and estimated depths for Co-60 buried in sand.

The experiment was also carried out using a 9-kBq Co-60 point source. Due to the low activity of the source, the scanning area was reduced to 20×20 cm^2^, while the scanning time was increased to 40 min per *x*-*y* position. The results for both the 1.77-MeV and 1.33-MeV photo-peaks of the Co-60 gamma spectrum for depths from 1 cm to 4 cm at 1-cm intervals are shown in Figure 10a,b. The similarity in the estimated depths from both peaks can be observed up to 3 cm, after which both graphs differ dramatically. This is not expected because gamma rays from both energy peaks have similar mass attenuation coefficients, i.e., 0.059 for 1.17-MeV gamma rays and 0.0552 for 1.33-MeV gamma rays. In addition, gamma rays from both energy peaks have the same probability of being emitted from the Co-60 nucleus. Due to these reasons, estimates after 3 cm were considered to be erroneous and were thus excluded from the linear fit between the real and estimated depths shown in Figure 10b. This maximum detectable depth of 3 cm achieved for 9-kBq Co-60 source buried in sand is a significant improvement over the technique reported in [12], which achieved a similar maximum depth limit for a 40-kBq Co-60 source also buried in sand. Finally, this result confirms that this method can be used both with other radionuclides and with low level buried wastes.

## 4. Conclusions

Improvements in the depth estimation results of the approximate 3D attenuation model using a CZT detector have been presented. The results showed that the model was able to non-intrusively estimate the depth of a 329 kBq Cs-137 radioactive source buried up to 18 cm in sand with a significantly lower average count rate of 14 cps compared to previous results of 12 cm with an average count rate of 100 cps. This will enable the rapid non-intrusive localisation of buried radioactive wastes. Furthermore, the results also confirmed that the depth limit depends on the data acquisition time. Therefore, increasing the data acquisition time will enable the estimation of the depth of wastes buried deeper in the sand. In addition, the result from the experiment using a 9-kBq Co-60 radioactive source confirmed that the model can be used with any gamma radiation source and is also capable of estimating the depth of buried sources with very weak activity. Furthermore, the explicit dependence of the model on the density of the material means that this method can be extended to any material, e.g., concrete, by substituting the material’s density in the model. Consequently, the method will find wide application in nuclear decommissioning, land remediation, nuclear security and non-proliferation activities. Finally, areas of further research include investigation of the method’s performance using non-point sources and multiple hot spots within the scanning area. These will further improve the robustness of this non-intrusive depth estimation method.

## Figures and Tables

**Table 1:** Elemental composition from SEM analysis of the sand used in the experiment.

Element	Weight Fraction
C	0.1714
O	0.5163
Na	0.0013
Al	0.0151
Si	0.2755
K	0.0072
Ca	0.0006
Fe	0.0072
P	0.0003
S	0.0004
Ti	0.0005
Cu	0.0009
Mg	0.0020
Zn	0.0014
	1.0000

**Table 2:** Parameters for the linear fit between the real and estimated depth from experiments and simulation.

	Depths (cm)	Gradient	Intercept
Experiment			
15 min	2–14	1.4 ± 0.1	−1.6 ± 0.9
20 min	2–16	1.4 ± 0.1	−1.5 ± 1.2
25 min	2–18	1.5 ± 0.2	−2.2 ± 1.6
**Simulation [13]**	2–40	1.6 ± 0.1	−2.0 ± 0.6
